# Efficacy of zinc sulfate on indirect hyperbilirubinemia in premature infants admitted to neonatal intensive care unit: a double-blind, randomized clinical trial

**DOI:** 10.1186/s12887-020-02025-9

**Published:** 2020-03-19

**Authors:** Gholamreza Faal, Hoda Khatib Masjedi, Gholamreza Sharifzadeh, Zahra Kiani

**Affiliations:** 1 Department of Pediatrics, Faculty of Medicine, Birjand University of Medical Sciences, South Khorasan Province, Birjand, Iran; 2 Faculty of Medicine, Birjand University of Medical Sciences, Birjand, Iran; 3 Faculty of Health, Birjand University of Medical Sciences, Birjand, Iran; 4 Faculty of Pharmacy, Birjand University of Medical Sciences, Birjand, Iran

**Keywords:** Jaundice, Preterm infants, Zinc sulfate

## Abstract

**Background:**

Hyperbilirubinemia is a common neonatal problem. Studies conducted on the effectiveness of zinc salts on serum indirect bilirubin levels in newborns have yielded different results, all calling for further research. This study aimed to determine the effect of oral zinc sulfate on indirect hyperbilirubinemia in preterm infants admitted to the neonatal intensive care unit.

**Methods:**

A randomized double-blind clinical trial was performed in the neonatal intensive care unit of Vali-e-Asr Hospital in Birjand, Iran. The study population comprised neonates aged between 31 and 36 gestational weeks, who required phototherapy in the neonatal intensive care unit. A total of 60 neonates were selected by census and allocated into an experimental group and a control group. In addition to phototherapy, the experimental group received 1 cc/Kg zinc sulfate syrup (containing 5 mg/5 cc zinc sulfate; Merck Company, Germany), and the control group received a placebo syrup (containing 1 cc/kg sucrose). Data were analyzed in SPSS-21 software using the independent t-test, repeated-measures ANOVA, Bonferroni post-hoc test, and Mann-Whitney test. *P*-values smaller than 0.05 were considered significant.

**Results:**

Bilirubin level changes in the experimental and control groups six hours after intervention were − 1.45 ± 3.23 and − 0.49 ± 0.37 (*p* = 0.024), respectively. The changes 24 and 48 h after intervention were-3.26 ± 2.78 and − 1.89 ± 1.20 (*p* = 0.017) in the experimental group and − 4.89 ± 2.76 and − 3.98 ± 2.32 (*p* = 0.23) in the control group, respectively. There was no significant difference in the phototherapy duration between the two groups (*p* = 0.24).

**Conclusions:**

The results of this study showed that the use of zinc sulfate syrup in preterm infants with indirect hyperbilirubinemia significantly reduced bilirubin levels within 48 h of treatment.

**Trial registration:**

Trial registration: IRCT, IRCT2015120825439N1. Registered 21 February 2016, http://irct.ir/trial/21277

## Background

Hyperbilirubinemia is a common and often benign neonatal problem. Jaundice is found in about 60% of term and 80% of preterm infants during the first weeks of life. The enterohepatic cycle, along with one of the following mechanisms, appears to increase serum bilirubin levels in infants: 1) decreased calorie intake, 2) increased lipid uptake, 3) decreased intestinal urobilinogene production, or 4) increased glucoronidase activity in breast milk users [1]. Overall, hyperbilirubinemia occurs in infants due to its increased production after the destruction of red blood cells. This is the primary mechanism of bilirubin excretion, whereby bilirubin is excreted in bile along with the stool [2–4].

Among the early lines of jaundice treatment is phototherapy, followed by blood exchange transfusion if there is no response to phototherapy or severe jaundice [5, 6]. Other treatments include the use of high-dose intravenous immunoglobulin [7], metalloporphyrins [8], and phenobarbital [9].

One of the methods used to treat indirect hyperbilirubinemia is to use a zinc solution [10]. Studies have shown that chronic or acute use of zinc salts can reduce serum bilirubin levels by inhibiting the enterohepatic cycle of indirect bilirubin [11]. The oral administration of zinc sulfate increases bilirubin excretion and decreases its serum level [3, 12].

Various studies have been conducted, mainly with term infants, to show different results as to the effect of zinc salts on the serum indirect bilirubin level, all calling for further research [3, 13]. Since the bilirubin excretion capacity in premature infants is less potent both in the liver and in the intestines than in term infants, we decided to investigate the effect of zinc sulfate in these infants. Therefore, this clinical trial was conducted to evaluate the efficacy of zinc sulfate on indirect hyperbilirubinemia in premature infants who were admitted to the neonatal intensive care unit.

## Methods

This study was a randomized, double-blind, clinical trial of 60 preterm infants suffering from jaundice as diagnosed during admission in the neonatal intensive care unit of Vali-e-Asr Hospital in Birjand, eastern Iran, from March to June 2016. Eligible participants were of a gestational age from 30 to 36 weeks and 6 days, and a weight range between 1500 and 2500 g.

Exclusion criteria included the need for intensive phototherapy and any clinical or laboratory evidence of hemolysis or infection, any congenital abnormality, dehydration, glucose 6-phosphate dehydrogenase deficiency (G6PD), ABO incompatibility, positive Coombs test, history of maternal phenobarbital intake, hypothyroidism, IUGR, mechanical ventilation, and the inability to be fed orally. To reject the exclusion criteria, a set of tests were performed, including the mother and infant blood group determination, hemoglobin, cell blood count, reticulocyte count, peripheral blood smear, Coombs test, and thyroid function and glucose 6-phosphate dehydrogenase tests.

The sample size was estimated as *n* = 30 subjects per group, based on the formula for comparing two means and the results of Rana et al.’s study [12] with X1 = 22.8, X2 = 35.6, S1 = 19.4, S2 = 16.1, α = 0.05, and β = 0.2 in each group of 30 subjects (Total number of subjects: *n* = 60). After the subjects were selected, one co-researcher allocated them into two groups via simple randomization, with one group receiving phototherapy plus zinc sulfate and the other group receiving phototherapy and placebo. The allocating co-researcher was not involved in the drug administration or assessments phases.

The study protocol was assessed by the faculty members of the pediatrics department at the faculty of medicine in terms of compliance with clinical and scientific standards. Prior to the implementation of the project, its protocol was approved at the University’s Ethics Committee on 31 Oct. 2015 (code IR.BUMS.1394.51). The protocol was also registered at the Iranian Registry of Clinical Trials on 21 Feb. 2016 (identifier: IRCT2015120825439N1).

The cons and pros of the intervention were explained to the patients’ legal guardians, and all of them signed written consent forms. Parents and legal guardians of infants were told that all infants would receive the usual treatment of hyperbilirubinemia, i.e., phototherapy. It was also explained that in the rare cases where zinc sulfate caused vomiting, diarrhea, and drug allergies, we would discontinue the medication and provide appropriate treatments, and if necessary, blood transfusions would be performed.

Parents and legal guardians of infants were told that all infants would receive the usual treatment of hyperbilirubinemia, i.e., phototherapy. It was also explained that in the rare cases where zinc sulfate caused vomiting, diarrhea, and drug allergies, we would discontinue the medication and provide appropriate treatments, and if necessary, blood exchange would be performed.

All infants received intensive phototherapy in line with the Clinical Practice Guideline Manual of the American Academy of Pediatrics [14]. Phototherapy was performed in both groups using a Tusan device equipped with four Philips lamps at a distance of 25 cm from the infant’s surface and at a minimum radiation intensity of 10 μW/cm2/nm.

In addition to phototherapy, the experimental group received 1 cc/Kg zinc sulfate syrup (containing 5 mg/5 cc zinc sulfate; Merck Company, Germany), and the control group received a placebo syrup (containing 1 cc/kg sucrose). To prepare the placebo syrup, 22.5 g sucrose was added to 450 cc distilled water and placed in the percolator to prepare the placebo syrup [5]. The placebo was identical with zinc sulfate in terms of volume, color, appearance, and packaging. A syringe was used for oral administration.

Zinc sulfate and placebo syrups were kept in the ward in bottles of similar color and shape labelled A and B, and were administered by nurses upon the prescription of the researcher. Blood samples were taken by nurses and sent to the laboratory. All who administered the medication and took blood samples as well as the laboratory staff were blind to the type of drug used. A checklist was used to collect infant information, including the type of drug used and bilirubin levels, which was filled out by one researcher and only s/he was aware of the course of treatment and the results.

The syrups were administered every 6 hours until the neonate did not require phototherapy, i.e., the total bilirubin level was less than 2 mg/dL below the phototherapy threshold [10]. Bilirubin levels were evaluated at baseline and 6 hours, 24 h, and 48 h after phototherapy. Direct, indirect, and total bilirubin levels were assessed in the University’s reference laboratory through the Diazo method of Pearlman and Lee using the Cobas Integra autoanalyzer. No interim analyses for efficacy or futility were performed.

The data were analyzed in SPSS software, version 19. Descriptive tests were used, including mean, standard deviation, frequency, and percentage. Moreover, data analysis was performed using independent t-test, repeated-measures ANOVA, post hoc Bonferroni test, and Mann-Whitney test. *P*-values smaller than or equal to 0.05 were considered significant.

**Fig. 1:**
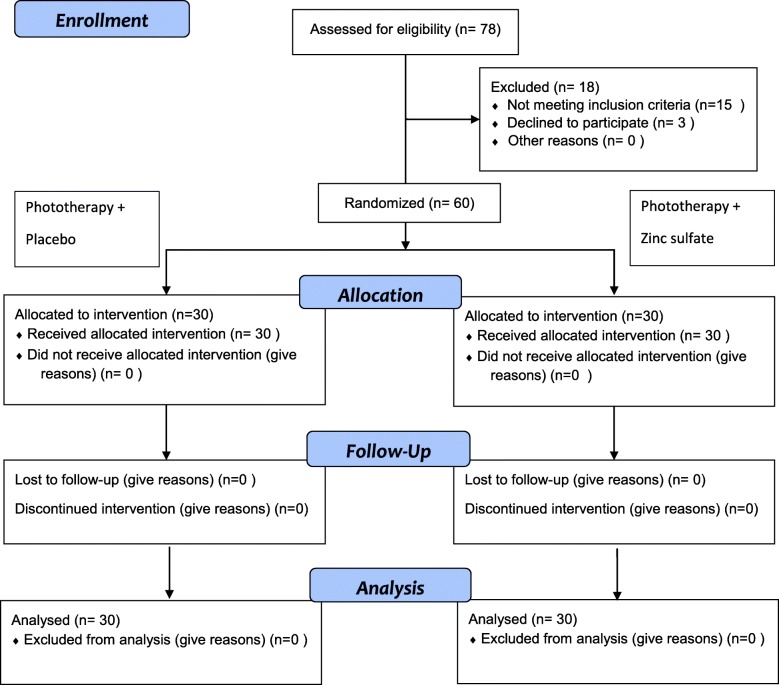
Flowchart of the study design

The flowchart of the study design is presented in Fig. 1.

After the clinical trial was registered, a total of 78 candidate neonates were identified in the NICU ward of Valiasr Hospital in Birjand within 4 months. Of them, 15 did not meet the eligibility criteria, and the guardians of three others did not agree with participation. Therefore, 60 subjects were recruited and randomly assigned into two groups, one receiving phototherapy plus zinc sulfate syrup and the other group receiving phototherapy and placebo.

The results showed that there was no significant difference between the groups in terms of sex, birth weight, chronological age, and gestational age (Table 1).

**Table 1:** Comparison of gender and birth weight distribution of neonates in experimental and control groups

Study group	Experimental Frequency (percentage)	Control Frequency (percentage)	*P*-value Chi-square test
Variable			
Birth weight (gr)			
< 1500	9 (30)	12 (40)	*P* = 0.065
≥ 1500	21 (70)	18 (60)	
Gender			
Female	11 (44)	14 (56)	*P* = 0.39
Male	14 (56)	11 (44)	
Study group	Experimental Mean ± Standard deviation ($\overline{\mathrm{X}}\pm SD\Big)$	Control Mean ± Standard deviation ($\overline{\mathrm{X}}\pm SD\Big)$	Independent t-test
Variable			
Neonate’s age (hour)	70.21 ± 8.9	65.20 ± 6.8	*P* = 0.35
Gestational age (week)	33.2 ± 1.27	32.1 ± 1.77	*P* = 0.06

Table 2 shows a summary of the results of the repeated measures ANOVA for baseline, six hours after the intervention, and 24 h after the intervention. According to the table, there was a significant difference between experimental and control groups before, six hours, and 24 h after the intervention. The mean bilirubin level in the experimental group decreased from 12.71 to 9.95 in the experimental group and from 10.35 to 8.44 in the control group.

**Table 2:** Comparison of mean bilirubin levels before, 6 h after, and 24 h after intervention in study groups

Study group	Experimental Mean ± Standard deviation ($\overline{\mathrm{X}}\pm SD\Big)$	Control Mean ± Standard deviation ($\overline{\mathrm{X}}\pm SD\Big)$	Independent t-test
Time point			
Before intervention (mg/dL) 6 h after intervention (mg/dL) 24 h after intervention (mg/dL)	12.2 ± 71.67	10.2 ± 35.24	
	11.2 ± 26.62	9.2 ± 86.32	
	9.2 ± 45.83	8.2 ± 44.10	
Repeated measures ANOVA result	*P* < 0.001		
Bonferroni post-hoc test result	Before and six hours after intervention (*p* = 0.004) Before and 24 h after intervention (*p* < 0.001) Six hours and 24 after intervention (*p* < 0.001)	Before and six hours after intervention (*p* = 0.001) Before and 24 h after intervention (*p* < 0.001) Six hours and 24 after intervention (p < 0.001)	

The independent t-test was used to compare the mean changes in bilirubin levels from baseline to 6, 24, and 48 h after the intervention. The results revealed a significant difference between the mean changes in bilirubin levels from baseline to six hours after the intervention from baseline to 24 h after the intervention in the two groups, with the average change being more considerable in the experimental group. However, there was no significant difference in bilirubin changes from baseline to 48 h after the intervention in the two groups (Table 3).

**Table 3:** Comparison of mean bilirubin level changes at baseline and the time points 6, 24, and 48 h after treatment in study groups

Study group	Experimental ($\overline{\mathrm{X}}\pm SD\Big)$	Control ($\overline{\mathrm{X}}\pm SD\Big)$	Independent t-test result
Bilirubin level			
Before and 6 hours after intervention	−1.3 ± 45.23	−0.0 ± 49.37	*P* = 0.024; t = 2.31
Before and 24 h after intervention	−3.2 ± 26.78	−1.1 ± 89.20	*P* = 0.017; t = 2.45
Before and 48 h after intervention	−4.2 ± 89.76	−3.2 ± 98.32	*P* = 0.23; t = 1.22

$\overline{\mathrm{X}}\pm SD$: Mean ± Standard deviation (mg/dL).

The results of the Mann-Whitney test showed no significant differences in the two groups in terms of the mean duration of phototherapy (*p* = 0.24; Table 4).

**Table 4:** Comparison of mean phototherapy duration in the experimental and control groups

Statistical index	Mean ± Standard deviation (mg/dL)
Group	
Experimental (hour)	42.18 ± 8.8
Control (hour)	12 ± 48.6
Mann-Whitney test result	*P* = 0.24; z = 1.17

No infants received additional treatment due to the effects of zinc sulfate consumption or exacerbation of jaundice.

## Discussion

This clinical trial was conducted to evaluate the effect of zinc sulfate on serum indirect bilirubinemia in preterm infants admitted to the neonatal intensive care unit. In both groups, serum bilirubin levels decreased significantly during treatment. The results of this study showed that the mean changes in bilirubin levels during the first six to 24 h of treatment in the experimental group was significantly greater than in the control group. During this time, the bilirubin level of the zinc recipient group had a significant reduction in the control group. There was no significant difference between the experimental and control groups concerning bilirubin level changes before and 48 h after the intervention and the mean phototherapy duration in the groups. None of the neonates had any side effects of zinc sulfate syrup.

Studies have, thus far, been conducted on the effects of zinc salts on neonatal jaundice. Along with the results of this study, regarding the significant reduction in bilirubin levels in the experimental group (treated with zinc syrup), Babaei et al. (2014) also found that 5 mg zinc sulfate daily consumption was associated with a reduction in serum bilirubin levels [15]. The results from Méndez-Sánchez et al.’s study (2002), consistent with the findings of this study, showed that the therapeutic use of zinc salts significantly reduced serum bilirubin levels in patients [11]. In Méndez-Sánchez et al.’s study, patients received a single 40 mg dose of zinc sulfate and another group received zinc sulfate at 100 mg every 24 h for seven days. The study showed that the acute or chronic use of zinc salts significantly reduced serum bilirubin levels in patients with Gilbert’s disease [11]. In a randomized, double-blind clinical trial, Mohammadzadeh et al. (2016) assessed the efficacy of zinc sulfate in reducing hyperbilirubinemia in low birth weight infants. They found that the administration of 10 mg zinc sulfate twice daily could significantly decrease bilirubin levels during the first 24 h of administration [16]. The theoretical explanation for this observation may concern with the physiological effects of zinc sulfate in the body. Zinc sulfate intake has been associated with an increase in the number of bowel movements, and this excretion, in turn, is likely to decrease the enterohepatic cycle, thereby reducing serum bilirubin levels [15].

Besides the physiologic effects of zinc sulfate on the regulation of bilirubin metabolism, studies have shown that children with hyperbilirubinemia have lower serum zink levels. In Beskabadi et al.’s study (2015), the mean serum zinc levels in healthy and hyperbilirubinemia newborns were 245.17 ± 1024.74 μmol/L and 241.17 ± 1024.74 μmol/L, respectively. The study concluded that higher levels of zinc had protective effects against hyperbilirubinemia [16].

Findings from some studies performed in this regard are not consistent with the results of this study, and in some cases, the use of zinc sulfate has not affected the bilirubin level in neonates. For example, findings from Mohammadzadeh et al.’s study [14], contrary to the findings of this study, showed that zinc had no apparent prophylactic effects. Also, Kumar et al.’s study [13] showed that there was no clinical benefit in using a zinc solution to treat neonatal jaundice. Differences in findings of studies thus far conducted can be attributed to the application of different doses and methods of prescribing the sulfate syrup as well as the differences in the overall approach adopted in studies (for neonates or for therapeutic / prophylactic use). Also, given the impact of zinc sulfate on the enterohepatic cycle of bilirubin, a longer administration of zinc sulfate at a higher dose, as well as its association with other zinc salts, can be contributory to reducing bilirubin levels [3, 17].

Regarding the association between the therapeutic use of zinc and the duration of phototherapy, Patton et al. stated that there was no significant difference in the duration of phototherapy between the two groups and that 5 mg twice daily use of sulfate had no significant effect on the duration of hyperbilirubinemia [11]. Consistent with this study, Kumar et al.’s study [18] showed that the duration of phototherapy did not differ significantly in the zinc and placebo groups [13]. Unlike our results, Hashemian et al.’s study (2014) found that the oral administration of zinc sulfate in jaundiced neonates may reduce the duration of phototherapy and, therefore, they recommended zinc syrup, along with phototherapy, as a safe and effective treatment for jaundice [19]. In Maamouri et al.’ study (2013), children in the placebo group were more in need of hospital admission and phototherapy treatment, and this study showed that due to the reduced hospitalization in patients receiving zinc sulfate and the shorter duration of phototherapy, it would be useful to administer this medication in these infants [3]. In Rana et al.’s study [12], also, the duration of phototherapy was significantly lower in the zinc group than in the placebo group [12]. It should be noted that in Maamouri et al. (2013) [3] and Rana et al.’s studies [12], the zinc sulfate syrup was used proactively in neonates to prevent the incidence of hyperbolic syndrome in a high-risk group. Therefore, the newborns consumed the zinc syrup since admission and underwent concurrent phototherapy in case of hyperbilirubinemia. Considering that prolonged use of this drug is associated with an increased risk of diarrhea and a reduction in enterohepatic cycle and thus a reduction in serum bilirubin levels, this seems to be the reason for the reduced duration of phototherapy. Mohammadzadeh et al. [16] assessed the efficacy of zinc syrup in reducing hyperlipidemia in low birth weight infants, with a significant decrease in bilirubin levels during the first 24 h.

Given the limited number of patients referred to the pediatrics ward, one limitation of the study is that it was not possible to have a larger sample size in this study. Hence, this project is limited in terms of the generalizability of the results to neonates of higher gestational age, as well as newborns with disorders associated with hyperbilirubinemia (such as ABO and Rh incompatibility). Another limitation of this study is the non-generalizability of its findings as concerns with the prophylactic effects of this treatment in preventing neonatal jaundice. In light of the positive results of this study on the efficacy of zinc sulfate syrup in decreasing premature neonates’ hyperbilirubinemia and the lack of obvious clinical complications, it is recommended that future research studies assess the impact of preventative use of this drug in populations with high-risk hyperbilirubinemia. It is also recommended to study the effectiveness of zinc sulfate on bilirubin reduction in other clinical subgroups, including infants of higher age or infants with disorders associated with hyperbilirubinemia.

## Conclusions

The results of this study showed that the use of zinc sulfate syrup for indirect hyperbilirubinemia in pre-term neonates was associated with a significant reduction in bilirubin levels within 24 h of administration. It is worth noting that the results of previous studies and the current one indicate that the zinc sulfate syrup is safe for use in infants and is not associated with complications such as vomiting and skin rash (except for the increased frequency of bowel movements). Therefore, it is recommended to use the zinc sulfate syrup (1 cc/kg) in preterm infants with indirect hyperlipidemia along with reasonable use of phototherapy.

## Data Availability

The datasets used and/or analyzed during the current study are available from the corresponding author on reasonable request.

## Abbreviations

ANOVA: Analysis of variance
IUGR: Intrauterine growth retardation
Rh: Rhesus
